# The Dynamic Relationship Between Objective and Subjective Housing Affordability and Mental Health

**DOI:** 10.1007/s11524-026-01068-0

**Published:** 2026-03-26

**Authors:** Geon Kim, Da Yoon Kim, Bo Kyong Seo

**Affiliations:** 1 Department of Urban Planning and Real Estate, Chung-Ang University, 84, Heukseok-Ro, Dongjak-Gu, Seoul, 06974 Republic of Korea; 2 Department of Urban Planning and Policy, University of Illinois at Chicago, 412 S. Peoria, 115 CUPPAH (MC 350), Chicago, IL 60607 USA; 3 Department of Applied Social Sciences, The Hong Kong Polytechnic University, Hung Hom, Hong Kong

**Keywords:** Objective affordability, Subjective affordability, Mental health, Stress, Depressive symptoms

## Abstract

This study explored how housing affordability is associated with mental health by focusing on the moderating role of subjective perceptions of rent burden. Drawing on data from the Korean Labor and Income Panel Study, we examined whether the association between unaffordable housing (assessed using the rent-to-income ratio) and mental health varied depending on the residents’ perceptions of housing cost burden. The findings revealed that mental health outcomes were poorer when high housing costs were accompanied by a sense of burden. These interaction patterns were especially pronounced among households in the bottom 40% of the income distribution, suggesting that affordability-related mental health risks were disproportionately experienced by lower-income groups. The results call for closer attention to subjective rent burden and its psychological consequences, as well as broader exploration of housing interventions and policies that improve residential environments to alleviate perceived housing stress.

**Supplementary Information:**

The online version contains supplementary material available at 10.1007/s11524-026-01068-0.

## Introduction

Housing affordability has steadily declined globally over the past few decades [1]. According to United Nations-Habitat data [2], 55% of the global population spends over 30% of their household income on housing, which is a widely accepted threshold for determining housing cost burden [3, 4]. The share of housing-related expenditures in household income also rose by 15% between 1995 and 2022, with a sharp increase since the mid-2000s [5]. More recently, many countries have faced housing affordability crises owing to limited housing supply and stagnant income increases, particularly in the wake of the coronavirus disease 2019 pandemic [6]. As housing costs usually account for the largest share of household expenditure, declining housing affordability likely challenges the well-being and mental health of households with economic constraints [7, 8]. Korea is no exception to the global trend in the challenges posed by housing affordability. Several studies have estimated that approximately 24–33% of private renter households in Korea spend more than 30% of their income on housing [9, 10].

Housing affordability is an important social determinant of mental health [11, 12]. The link between housing affordability and mental health and its mechanisms have been well-documented [3, 11–14]. Prior studies have demonstrated that spending too much on housing or having too little income for non-housing expenses is likely to negatively impact psychological well-being, particularly in lower-income groups [3, 10]. Socioeconomically disadvantaged households in cities with high housing price-to-income ratios demonstrate lower levels of subjective well-being than those with more affordable ratios [15]. The adverse mental health effects of unaffordable housing are more pronounced among renters than homeowners, as well as among younger people than among older people, arguably because of their precarious tenure and financial status [12, 13, 16, 17]. Moreover, insufficient after-housing income has been found to compromise household consumption of other basic necessities, such as food and medications, leading to mental distress of financially constrained families [8].

Given the evident association between unaffordable housing and poor mental health, we aimed to refine the existing methodological approaches to understanding this relationship by examining the moderating role of subjective affordability as a new dimension. How “unaffordable” housing is defined is a critical issue, as this definition essentially informs the identification of target groups for policies intended to help reduce mental health risks induced by housing affordability problems. In the mental health literature, most studies have used objective indicators to measure housing affordability. While several studies have concerned whether after-housing income is sufficient for a household's basic needs [8, 14, 18] or whether a household has experienced rent arrears [19], the most widely used indicator focuses on whether a household pays 30% (sometimes ranging from 25% up to 50% depending on income levels) or more of its income on housing, known as a ratio approach [20, 21]. However, whether this ratio approach is the best tool to capture households’ experiences of affordability stress, which likely influences mental health, remains debatable. Although psychological hardships among low-income families paying over 30% of their income on housing were found to be more severe than those in middle- and upper-income groups [3, 11, 13, 17], the ratio-based thresholds are considered a somewhat arbitrary method of defining unaffordable housing [4] and provide little account of how objective housing affordability is subjectively and psychologically experienced by the occupants [21].

The importance of integrating subjective social indicators with objective indicators to elucidate humans’ well-being has been gradually recognized since the 1970 s [21, 22]. Further, measuring individuals’ perceptions can adequately reflect their experiences of certain conditions, thus providing a more complete picture of what objective indicators alone cannot present [23]. In this context, the present study examined the role of subjective housing affordability (i.e., perceived housing cost burden) on the association between objective housing affordability (i.e., ratio approach) and mental health.

There are two primary reasons underlying our hypothesis. First, objective and subjective housing affordability measures do not necessarily capture the same underlying concepts. Heylen [21] found that only less than 30% of households show a match between objective and subjective housing affordability. Seo et al. [24] also found that approximately 40% of their study sample exhibited a mismatch between their objective rental affordability status (i.e., affordable versus unaffordable according to the 30% rent-to-income ratio [RIR] standard) and their perceived rent burden, and this incongruence was more pronounced in the bottom 40% income group. In other words, some people paying less than 30% of their income for housing might still feel housing cost burdens, and hence, have poor mental health. Conversely, some people who pay more than the specified threshold ratio for housing may not perceive housing cost burdens and, therefore, may not feel stressed. In the classification system proposed by Noll [22], the former is classified as the “dissonance” group, and the latter as the “adaptation” group (see Seo et al. [24] for a more complete description of the mismatch between objective and subjective housing affordability and possible underlying mechanisms).

Second, a growing body of research has identified a close connection between subjective housing factors and residents’ well-being, at times finding a better fit of subjective housing indicators, compared to objective ones, in explaining psychological status. For example, Pollack et al. [25] found that perceived difficulty in affording housing costs is significantly associated with self-reported health outcomes. Moreover, Xie et al. [26] suggested that subjective assessments of housing (e.g., housing satisfaction) explain the variance in mental health outcomes better than objective assessments (e.g., housing quality and tenure) do. Similarly, Zhang et al. [27] found that mental health was influenced by the subjective neighborhood environment more than by objective neighborhood characteristics. Acolin and Reina [28] also demonstrated that people paying more than 30% of their household income for housing (objectively unaffordable) but feeling no burden (subjectively affordable) showed a higher level of life satisfaction than those paying less than 30% (objectively affordable) but feeling a heavy burden (subjectively unaffordable). They found that the negative association between perceived burden and life satisfaction was stronger among lower income groups. In mental health research, subjective socioeconomic status is more strongly associated with mental health than objective measures [27, 28]. Prior studies also suggest that subjective housing attributes moderate the association between objective environments and mental health [29, 30]. If our study further supports the relevance of subjective housing affordability for mental health, and it is shown to modify the relationship between objective housing affordability and mental health, our findings may help motivate refinements to existing approaches for measuring housing affordability in mental health research by encouraging the integration of both objective and subjective indicators.

Accordingly, in the present study, we specifically examined the following research questions (RQs):

- RQ1: Does subjective housing affordability moderate the relationship between objective affordability and mental health?
- RQ2: Does this relationship differ across different income groups?

It should be noted that the purpose of this study was not to negate the mental health effects of objectively measured housing affordability but rather to advance current knowledge of how mental well-being is shaped by housing affordability, measured from different angles. The findings of this study will contribute to the redefinition of the groups exposed to housing affordability-related mental health risks.

## Methods

### Data

This study draws on data from the 2022 and 2023 waves of the Korean Labor and Income Panel Study (KLIPS). Conducted by the Korea Labor Institute, a government-funded research organization, KLIPS is a nationally representative longitudinal survey that has been collecting data since 1998. KLIPS tracks the same households and individuals annually, periodically adding refreshment samples (most recently in 2009 and 2018) to compensate for attrition and preserve representativeness. The survey provides detailed information on the socioeconomic characteristics of urban Korean households and their members, including income, housing tenure and type, educational attainment, and employment status. The survey employed a systematic sampling framework and appropriate weights were applied to account for the survey design and ensure national representativeness.

The KLIPS core questionnaire remained largely consistent across the waves, whereas supplemental modules covering different thematic areas were administered annually. In 2023, an additional health module was included to collect information on physical activity, self-rated health, and health service utilization. This study utilized this supplemental health survey to examine the relationship between housing affordability and mental health as measured by levels of stress and depressive symptoms. To strengthen the validity of the causal interpretations, we used the 2022 data points for independent variables and 2023 data for outcome variables. A more detailed rationale for this approach is provided in Sect. "Analytic Strategy" below.

The analytical sample was restricted to private renters. Renters differ from homeowners in terms of housing cost structures; homeowners often pay mortgage interest and property taxes, whereas renters pay regular rent. Moreover, renters face unique stressors, such as lease renewal uncertainty and exposure to rent fluctuations, which may increase their sensitivity to stress and depressive symptoms [31]. Given these distinctions, this study exclusively focused on renters.

### Measures

#### Measures of Mental Health

To assess mental health outcomes, we used two self-reported indicators: perceived stress and depressive symptoms. In the KLIPS, the head of household was asked to evaluate their psychological condition using a 10-point Likert scale. Stress was measured using the question, "Over the past two weeks, how much stress have you experienced in your daily life?" The response scale ranged from 1 ("Very low") to 10 ("Very high"), with higher scores indicating greater levels of perceived stress. Depressive symptoms were assessed using the question, "Over the past two weeks, how often have you felt sad or depressed in your daily life?" This item also used a 10-point Likert scale, where 1 indicates "Not at all depressed" and 10 indicates "Always depressed."

Although the mental health module in the KLIPS did not include a comprehensive diagnostic instrument such as the Depression Anxiety Stress Scales-21 (DASS-21), it provides a rare opportunity to examine self-reported mental health alongside both objective and subjective measures of housing affordability within a nationally representative dataset.

#### Measures of Housing Affordability and Moderator

Housing affordability was measured using objective and subjective indicators. As an objective measure, we calculated the ratio of monthly rent to reported monthly household income. Consistent with prior studies on housing affordability [3, 20], households spending 30% or more of their income on rent were coded as 1 (objectively unaffordable), and those below this threshold were coded as 0 (objectively affordable). For the subjective measure, we used a self-report item asking respondents whether they perceived their housing costs as burdensome. To reduce confusion, we referred to this variable as *subjective unaffordability*: respondents who reported feeling burdened were coded as 1 (*subjectively unaffordable*), whereas all others were coded as 0 (*subjectively affordable*).^1^ Using these two variables, we constructed an interaction term to serve as a key independent variable in our model. This allowed us to examine whether subjective affordability moderates the relationship between objective affordability and mental health outcomes and whether such moderation differs across income groups.

#### Covariates

Various socioeconomic characteristics were included as covariates in the regression models. These variables were selected based on prior research and theoretical considerations regarding potential confounding factors in the relationship between housing affordability and mental health [24, 29].^2^

First, a binary variable was constructed for residences in the Seoul metropolitan area, which included Seoul, Incheon, and Gyeonggi Province. Households located in this region were coded as 1, and those outside were coded as 0. The Seoul metropolitan area is home to more than half of South Korea's population and employs approximately 50% of its workforce [32]. Prior studies have shown that living in this region is associated with distinct patterns of housing affordability, stress, and life satisfaction compared to living in non-metropolitan areas [33].

In addition, several household- and housing-level characteristics were adjusted. Educational attainment was coded as 1 if the head of the household had completed education beyond high school and 0 otherwise. Age was included as a continuous variable. Marital status was coded as 1 if the respondent was married, and 0 otherwise. Sex was coded as 1 for female and 0 for male. We also included the number of children and total number of household members as continuous covariates. Finally, housing type was included as a binary variable, coded as 1 if the household resided in an apartment, the predominant housing type in South Korea, and 0 otherwise [34].

**Fig. 1:**
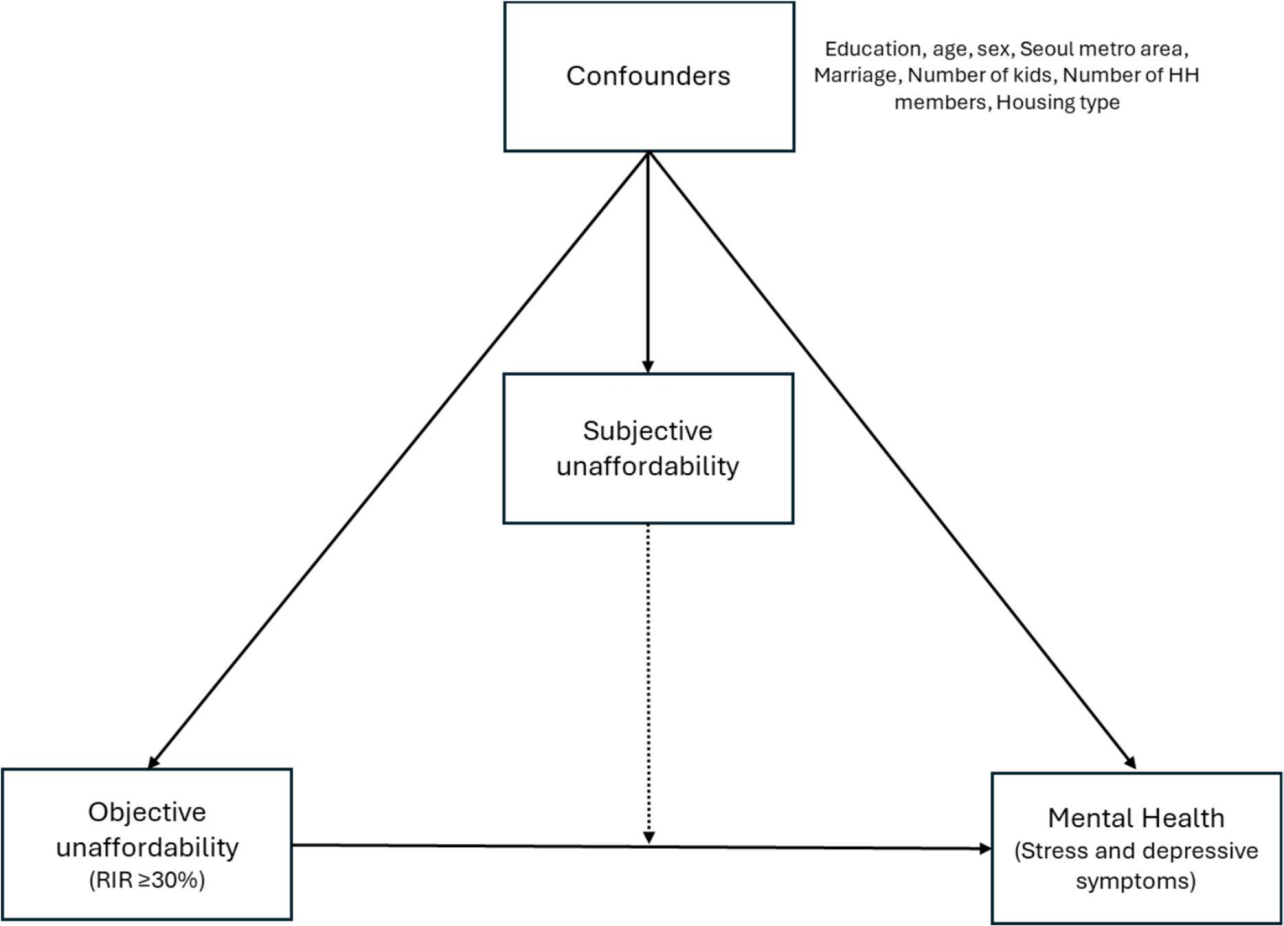
Conceptual model of housing affordability and mental health

Figure 1 presents the variables discussed above and their relationships within the conceptual model of the study. It further illustrates the framework guiding the analysis and highlights the moderating role of subjective housing unaffordability in the association between objective housing costs and mental health outcomes.

### Analytic Strategy

To examine the potential moderating effect of subjective affordability on mental health outcomes, we included an interaction term between objective and subjective housing affordability. We used temporally lagged independent and dependent variables; mental health outcomes (stress and depressive symptoms) were drawn from the 2023 wave of the KLIPS, while all independent variables, including housing affordability measures and covariates, were taken from the 2022 wave to support a more rigorous temporal alignment of variables. In other words, we established a temporal ordering that helps to mitigate concerns about reverse causation and aligns with prior literature suggesting that housing conditions exert lagged effects on mental health [21]. This approach may help address the concerns of reverse causality, namely, the difficulty of disentangling whether unaffordable housing leads to higher levels of stress or depressive symptoms, or whether individuals experiencing such mental health challenges have a higher likelihood of perceiving their housing as unaffordable or encounter financial strain, although it does not fully eliminate these concerns. One limitation of this approach is that it does not capture changes in housing conditions that may have occurred between the two survey waves. To address this, we restricted our main analytical sample to individuals who did not relocate between 2022 and 2023, thereby increasing the consistency of housing-related exposure across the study period.

Next, we conducted subgroup analyses based on the income strata. Subgroup analyses (Tables 4 and 5) involved stratified models estimated separately for the bottom 40% and top 60% income groups. Rental unaffordability tends to disproportionately influence low-income households, which are also the primary targets of housing affordability policy interventions [17]. Prior research suggests that the relationship between affordability and mental health may vary across income levels [35]. Considering this, we divided the sample into the bottom 40% and top 60% of the household income distribution and compared these subgroup results with those from the full sample.

Although many studies define the objective affordability measure using the conventional 30% RIR threshold, this cutoff is widely acknowledged as a rule of thumb rather than a definitive standard. Thus, to test the robustness of our findings, we re-estimated the models using alternative thresholds of 25%, 35%, and 50% to define objective unaffordability.

All the data used in the regression analyses were weighted to reflect the complex sampling design and ensure generalizability to the national population. Descriptive statistics including means and frequencies were calculated using unweighted data to preserve the observed distribution characteristics of the sample.^3^ All analyses were conducted using R statistical software (version 4.4.3).

## Results

### Analysis Results for the Full Sample

Tables 1 and 2 present summary statistics of the key variables. Table 1 compares the respondents' characteristics between the objectively affordable and unaffordable groups. As expected, the proportion of individuals reporting subjective unaffordability was higher among those in the objectively unaffordable group compared to those in the objectively affordable group who still perceived their housing costs as burdensome. In terms of mental health outcomes, stress levels were similar between the two groups, whereas the objectively unaffordable group experienced slightly more depressive symptoms. Additionally, those in the unaffordable group were more likely to reside in the Seoul metropolitan area or apartments, and they were slightly older and lived in smaller households. 

Table 2 compares the subjectively affordable and unaffordable groups. As anticipated, the proportion of respondents spending more than 30% of their income on rent was higher among those who perceived housing costs as burdensome. In terms of mental health, individuals in the subjectively unaffordable group reported slightly higher levels of stress and depressive symptoms, although the differences were modest. Please place Table 1 and Table 2 here, immediately above "Next".

**Table 1:** Summary statistics by objective affordability

Variable	Objectively Affordable (RIR ≥ 30)	Objectively unaffordable (RIR < 30)	*P*-value
	(N = 2,414)	(N = 541)	
Subjective unaffordability	0.39 (0.49)	0.47 (0.50)	0.001
Stress	4.33 (1.82)	4.36 (1.97)	0.708
Depression	3.24 (1.93)	3.68 (1.97)	0.000
Seoul metropolitan area	0.54 (0.50)	0.71 (0.46)	0.000
Education	0.23 (0.42)	0.26 (0.44)	0.191
Age	56.29 (15.77)	63.15 (16.55)	0.000
Marriage	0.84 (0.37)	0.87 (0.34)	0.138
Sex	0.32 (0.47)	0.44 (0.50)	0.000
Number of kids	0.43 (0.81)	0.35 (0.72)	0.020
Number of household members	2.32 (1.29)	1.99 (1.20)	0.000
Housing type	0.46 (0.50)	0.56 (0.47)	0.000

The data are presented as mean (standard deviation). The variables were coded as follows: subjective unaffordability = 1 if housing costs were perceived as burdensome and 0 otherwise; Education = 1 if the education level was beyond high school, 0 if high school or less; Marriage = 1 if married and 0 otherwise; Sex = 1 for female and 0 for male; Housing type = 1 if living in an apartment, 0 otherwise. P-values were obtained from two-sample comparisons of means or proportions. RIR, rent-to-income ratio

**Table 2:** Summary statistics by subjective affordability

Variable	Subjectively affordable	Subjectively unaffordable	*P*-value
	(N = 1,754)	(N = 1,201)	
Objective unaffordability (RIR ≥ 30)	0.16 (0.37)	0.21 (0.41)	0.001
Stress	4.28 (1.84)	4.41 (1.86)	0.066
Depression	3.20 (1.94)	3.50 (1.95)	0.000
Seoul metropolitan area	0.56 (0.50)	0.57 (0.49)	0.596
Education	0.28 (0.45)	0.17 (0.37)	0.000
Age	55.55 (16.00)	60.45 (15.88)	0.000
Marriage	0.85 (0.36)	0.84 (0.37)	0.391
Sex	0.30 (0.46)	0.41 (0.49)	0.000
Number of kids	0.51 (0.85)	0.27 (0.68)	0.000
Number of household members	2.40 (1.30)	2.06 (1.21)	0.000
Housing type	0.49 (0.50)	0.46 (0.50)	0.047

The data are presented as mean (standard deviation). The variables were coded as follows: subjective unaffordability = 1 if housing costs were perceived as burdensome and 0 otherwise; Education = 1 if the education level was beyond high school, 0 if high school or less; Marriage = 1 if married and 0 otherwise; Sex = 1 for female and 0 for male; Housing type = 1 if living in an apartment, 0 otherwise. P-values were obtained from two-sample comparisons of means or proportions. RIR, rent-to-income ratio

Next, we examined the associations between housing affordability indicators and mental health outcomes.^4^ Table 3 presents the regression results for the entire sample. In Models 1 and 3, after adjusting for covariates, objective affordability was not significantly associated with stress or depressive symptoms. In contrast, individuals who perceived their housing costs as burdensome reported significantly higher levels of both stress and depressive symptoms, on average.

However, the effect of objective affordability on stress varied depending on subjective affordability. In Model 2, the interaction term was positive and statistically significant (0.597, *p* < 0.01). In other words, among renters who both had objectively unaffordable rents and were subjectively burdened, stress levels were 0.328 points (i.e., − 0.269 + 0.597) higher than those who had objectively unaffordable rents but did not feel burdened. Similarly, renters experiencing both high objective and subjective rent burdens reported depressive symptoms that were 0.191 points higher (i.e., − 0.220 + 0.411). These findings suggest that the combination of objective unaffordability and perceived burden is associated with significantly worse mental health outcomes, both in terms of stress and depressive symptoms, compared with either factor alone.

**Fig. 2:**
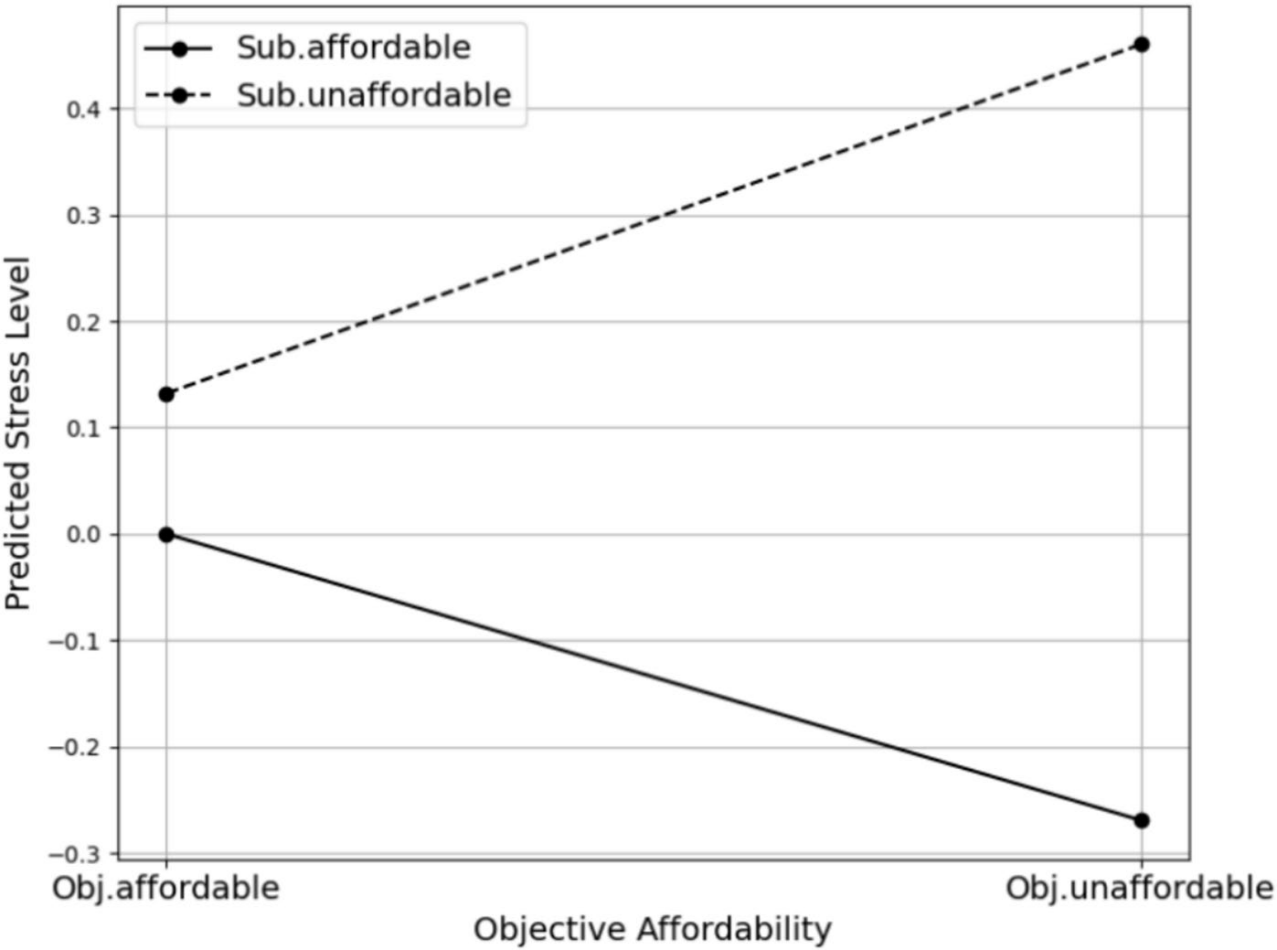
The interaction between objective and subjective affordability on stress

**Fig. 3:**
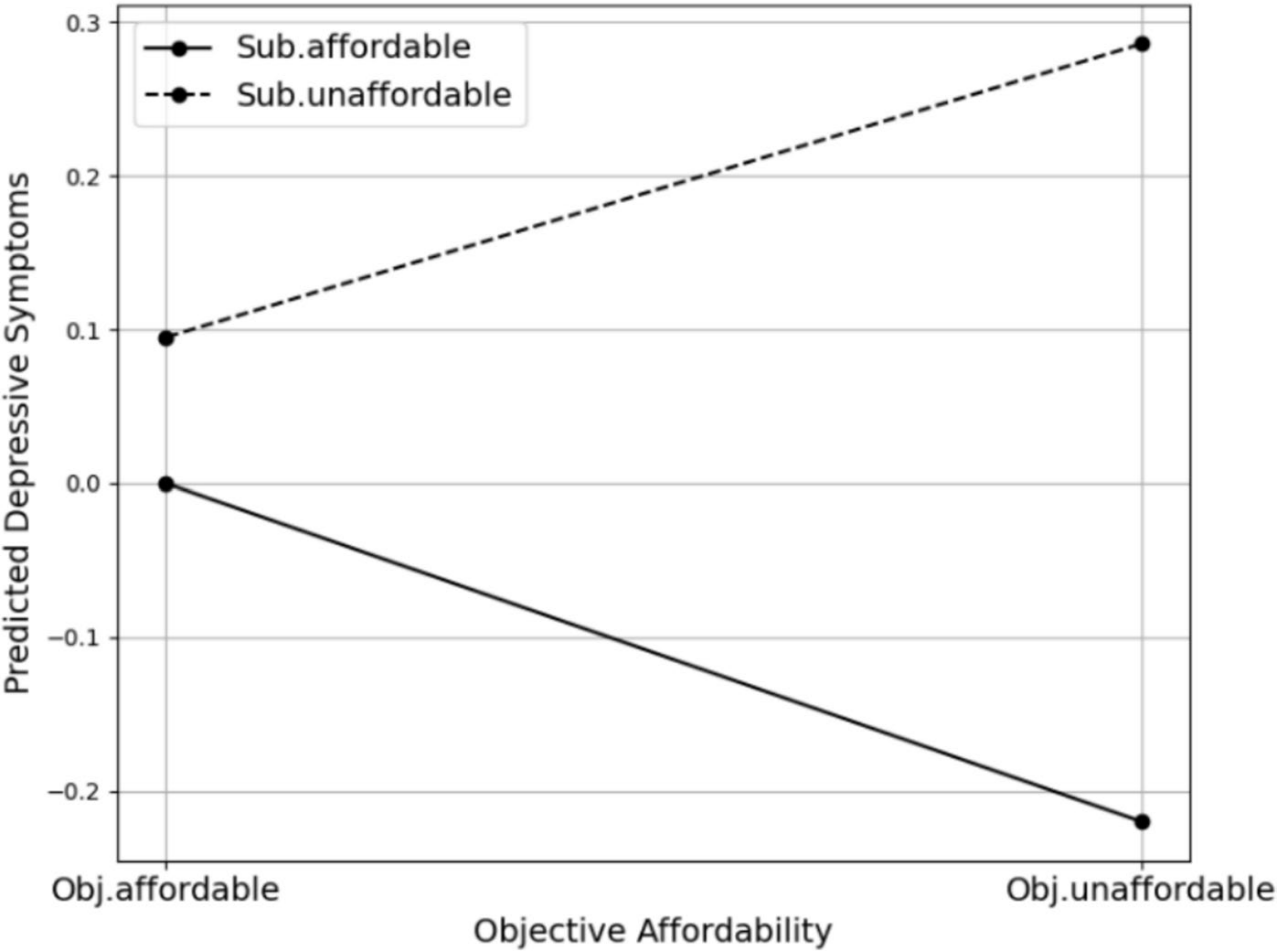
The interaction between objective and subjective affordability on depressive symptoms

These patterns were clearly illustrated in the interaction plots. The plotting of the interaction terms (Fig. 2) revealed that, in terms of stress, respondents who perceived their housing costs as unaffordable reported higher stress levels regardless of objective affordability. Moreover, among those facing objectively unaffordable housing costs, stress levels increased significantly when they perceived their rent as burdensome. A similar pattern emerged for depressive symptoms, although the effect was less pronounced (Fig. 3). While the interaction term was less pronounced than for stress, respondents who experienced both objective and subjective unaffordability tended to report elevated levels of depressive symptoms.

**Table 3:** Associations between housing affordability and mental health

Variable	Stress		Depressive symptoms	
	Model 1	Model 2	Model 3	Model 4
Objective unaffordability	0.006 (0.094)	− 0.269** (0.126)	− 0.030 (0.094)	− 0.220* (0.127)
Subjective unaffordability	0.254*** (0.075)	0.132 (0.084)	0.179** (0.075)	0.095 (0.084)
Seoul metropolitan area	− 0.114 (0.075)	− 0.091 (0.075)	− 0.089 (0.075)	− 0.073 (0.075)
Education	− 0.030 (0.086)	− 0.053 (0.086)	− 0.011 (0.087)	− 0.027 (0.087)
Age	− 0.007** (0.003)	− 0.006** (0.003)	0.027*** (0.003)	0.027*** (0.003)
Marriage	0.174 (0.130)	0.165 (0.130)	− 0.071 (0.131)	− 0.077 (0.131)
Sex	− 0.107 (0.084)	− 0.123 (0.084)	0.382*** (0.085)	0.371*** (0.085)
Number of kids	− 0.147** (0.067)	− 0.132** (0.067)	0.006 (0.067)	0.017 (0.067)
Number of household members	0.096** (0.045)	0.090** (0.045)	− 0.024 (0.045)	− 0.028 (0.045)
Housing type	− 0.301*** (0.076)	− 0.288*** (0.076)	− 0.382*** (0.076)	− 0.373*** (0.076)
Objective × subjective affordability		0.597*** (0.184)		0.411** (0.185)
Constant	4.719*** (0.218)	4.781*** (0.219)	1.659*** (0.219)	1.702*** (0.220)
Observations	2,955	2,955	2,955	2,955
Adjusted R^2^	0.011	0.014	0.068	0.069

*p < 0.1; **p < 0.05; ***p < 0.01. The robust standard error is shown in parentheses

The goodness of fit of the models was evaluated using the adjusted R-squared value, which ranged from 0.011 to 0.069. Although these values were relatively low, such levels of explained variance are not uncommon in studies focusing on mental health outcomes [26, 36].

### Results by Income Group

Next, we examined whether the association between housing affordability and mental health varied according to income level. Because psychological hardship is more likely to lead to material hardship, housing instability, and greater reliance on public assistance among low-income households, the association between housing unaffordability and mental health has heightened policy relevance in this group [37–39]. Consequently, we stratified analyses by income level to examine whether the patterns differed for those in the bottom 40% and top 60% of the income distribution (Tables 4 and 5).

**Table 4:** Association between affordability and mental health for the bottom 40% income group

Variable	Stress		Depressive symptoms	
	Model 1	Model 2	Model 3	Model 4
Objective affordability	− 0.017 (0.131)	− 0.270 (0.194)	− 0.124 (0.138)	− 0.376* (0.204)
Subjective affordability	0.167 (0.115)	0.041 (0.136)	0.021 (0.121)	− 0.105 (0.143)
Seoul metropolitan area	− 0.344*** (0.117)	− 0.328*** (0.118)	− 0.255** (0.124)	− 0.239* (0.124)
Education	0.310* (0.161)	0.277* (0.162)	− 0.136 (0.169)	− 0.168 (0.170)
Age	− 0.013*** (0.005)	− 0.013*** (0.005)	0.017*** (0.005)	0.018*** (0.005)
Marriage	0.613*** (0.189)	0.603*** (0.188)	0.149 (0.198)	0.139 (0.198)
Sex	− 0.311*** (0.119)	− 0.330*** (0.120)	0.146 (0.125)	0.128 (0.126)
Number of kids	− 0.201 (0.201)	− 0.165 (0.202)	− 0.081 (0.212)	− 0.045 (0.213)
Number of household members	0.189** (0.093)	0.181* (0.093)	0.138 (0.098)	0.130 (0.098)
Housing type	− 0.317*** (0.116)	− 0.314*** (0.116)	− 0.101 (0.123)	− 0.097 (0.122)
Objective × subjective affordability		0.451** (0.206)		0.449* (0.269)
Constant	5.096*** (0.352)	5.163*** (0.354)	2.422*** (0.370)	2.489*** (0.372)
Observations	1,150	1,150	1,150	1,150
Adjusted R^2^	0.040	0.041	0.030	0.032

*p < 0.1; **p < 0.05; ***p < 0.01. The robust standard error is shown in parentheses

**Table 5:** Association between affordability and mental health for the top 60% income group

Variable	Stress		Depressive symptoms	
	Model 1	Model 2	Model 3	Model 4
Objective affordability	− 0.152 (0.173)	− 0.295 (0.207)	− 0.154 (0.168)	− 0.164 (0.201)
Subjective affordability	0.181* (0.103)	0.143 (0.107)	0.136 (0.100)	0.133 (0.105)
Seoul metro area	− 0.031 (0.099)	0.032 (0.099)	0.064 (0.097)	0.064 (0.097)
Education	− 0.219** (0.107)	− 0.218** (0.107)	− 0.007 (0.104)	− 0.007 (0.104)
Age	− 0.001 (0.005)	− 0.001 (0.005)	0.027*** (0.005)	0.027*** (0.005)
Marriage	− 0.217 (0.187)	− 0.215 (0.187)	− 0.115 (0.182)	− 0.115 (0.182)
Sex	− 0.020 (0.125)	− 0.020 (0.125)	0.414*** (0.121)	0.414*** (0.121)
Number of kids	− 0.044 (0.081)	− 0.042 (0.081)	0.026 (0.079)	0.026 (0.079)
Number of household members	0.080 (0.061)	0.081 (0.061)	0.003 (0.059)	0.004 (0.059)
Housing type	− 0.250** (0.102)	− 0.243** (0.102)	− 0.503*** (0.099)	− 0.503*** (0.099)
Objective × subjective affordability		0.457 (0.363)		0.034 (0.353)
Constant	4.609*** (0.309)	4.609*** (0.309)	1.470*** (0.301)	1.470*** (0.301)
Observations	1,724	1,724	1,724	1,724
Adjusted R^2^	0.008	0.008	0.046	0.046

*p < 0.1; **p < 0.05; ***p < 0.01. The robust standard error is shown in parentheses

In the bottom 40% of the income group, the interaction terms were positively associated with both stress (0.451, *p* < 0.05) and depressive symptoms (0.449, *p* < 0.1), suggesting that the combination of objective and subjective unaffordability had a particularly detrimental impact on mental health in this group. These results indicate that, for low-income households, the psychological toll of unaffordable housing is especially severe when high rent payments are accompanied by a strong perception of burden. In contrast, no significant interaction was observed among the top 60% of the income group, indicating that the interaction observed in the full sample models (Table 3) may be largely influenced by the lower-income respondents. The magnitude of the interaction term for depressive symptoms differed substantially between income groups, with larger coefficients in the lower-income group.

### Robustness Check

We made several methodological decisions throughout the course of this analysis. First, to address the arbitrariness of relying on a single RIR cutoff of 30%, we estimated alternative specifications with thresholds set at 25%, 35%, and 50%. All findings remained robust across these alternative thresholds. The interaction between objective and subjective unaffordability increased in magnitude and statistical significance when using thresholds of 35% and 50%, which are commonly considered indicative of severe housing cost burden (Tables S5-S9 of the Supplementary materials).

Second, post-hoc power analyses were conducted to evaluate statistical power. The results supported our interpretation of the differential moderating role of subjective unaffordability by income level, despite limitations in statistical power (Table S10 of the Supplementary materials).

Third, instead of the primary income stratification approach that divided the sample into the bottom 40% and top 60% of the income distribution, we conducted supplementary analyses using an alternative three-group classification: the bottom 20%, middle 60%, and top 20%. The findings indicate that the interaction terms were most pronounced among the bottom 20% income group, remained statistically significant but attenuated in magnitude among the middle 60%, and became statistically insignificant among the top 20% (Tables S11-S13 of the Supplementary materials). This analysis provides consistent evidence that the moderating role of subjective affordability is more pronounced among lower-income groups.

Fourth, we conducted a supplemental analysis including all renters, regardless of their relocation status, to address concerns that excluding movers may introduce selection bias, given that housing cost–burdened households are more likely to experience involuntary moves that may adversely influence mental health (Tables S14-S16 of the Supplementary materials). Including movers substantially increased the sample size; however, the interaction between objective and subjective unaffordability became attenuated and statistically insignificant. We cautiously interpreted this attenuation as follows: because movers’ housing affordability in 2022 may no longer reflect their housing conditions in 2023, the risk of exposure misclassification is high, potentially biasing estimates toward the null. Importantly, this pattern does not contradict our main findings but is instead consistent with the measurement error introduced by changes in housing circumstances among movers.

## Discussion

This study examined the association between rental affordability and mental health and highlights the moderating role of residents' perceptions of rent burden. The interaction between objective and subjective unaffordability indicates that stress and depressive symptoms may be particularly high when individuals face high rent burdens and simultaneously perceive their housing costs as burdensome.

The above-mentioned pattern could be explained by at least three potential mechanisms. First, high housing costs can arise from rational and voluntary decisions such as prioritizing residential satisfaction; for example, choosing to pay higher rent in exchange for access to better schools or proximity to work. In such cases, satisfaction with one's residential environment may mitigate the perceived financial burden, thereby attenuating the negative effects of housing costs on mental health. However, when individuals are dissatisfied with their residential environment and perceive their rent as burdensome, the relationship between housing affordability and mental health becomes detrimental [17]. These findings expand on previous studies by Seo et al. [24] and Bentley et al. [35], who examined the interplay among income, housing affordability, and satisfaction with residential environments.

Another plausible explanation is psychological adaptation to high housing costs. According to adaptation theory [40], individuals may adjust to persistently high rent levels over time, reducing the likelihood of feeling subjectively burdened. This pattern is also consistent with the hedonic treadmill theory [41], which suggests that psychological well-being tends to return to baseline after negative events such as a rent increase.

A third possible mechanism is that renters with a high income or substantial assets may not perceive high housing costs as burdensome or may experience fewer adverse mental health effects. This aligns with the stress-buffering hypothesis, suggesting that economic resources can lessen the impact of stressors on mental health by enabling more effective coping, lowering perceived threats, and expanding the options available to manage challenging situations [42]. Consequently, renters with greater financial means may be less vulnerable to resource depletion, which may decrease their level of stress and increase their level of mental well-being. Conversely, renters with low income or minimal assets may perceive even moderate or normally expected” housing costs as burdensome, owing to their overall constrained financial circumstances. They may experience amplified psychological impacts despite facing objectively comparable levels of housing costs. Nevertheless, our data do not allow us to directly confirm the underlying mechanisms, such as psychological adaptation, hedonic adjustment, and stress buffering. As such, these mechanisms are best understood as interpretive frameworks that warrant direct empirical testing in future research.

Our findings also suggest that declines in mental health associated with poor housing affordability are concentrated among individuals in the bottom 40% of the income distribution, a pattern consistent with previous studies [3, 24]. The mechanisms underlying this association may differ significantly between the bottom 40% and higher-income groups. For example, limited financial resources may force difficult tradeoffs, where high rent payments displace essential spending on items such as food, healthcare, or education, directly contributing to psychological strain. In addition, while some degree of psychological adaptation to housing cost burdens is possible, it is plausible that many low-income participants have already surpassed the threshold at which the burden becomes overwhelming and inescapable. Furthermore, the lack of residential mobility in this group may result in more constrained involuntary housing choices, which can intensify the perceived burden and amplify negative mental health outcomes. In contrast, the absence of a significant association between poor housing affordability and mental health among the top 60% income group may reflect greater financial resilience, more robust coping strategies, or the fact that higher housing costs in this group are more often the result of intentional and voluntary decisions, making them less likely to be perceived as burdensome and less detrimental to mental health.

The patterns we observed offer several broad implications. Consistent with prior research on neighborhood quality and mental health, improvements in residential environments, spanning safety, amenities, and social support, may help reduce the perceived housing burden among low-income renters [29, 30], in part because higher-quality environments make rent feel more justified and therefore less burdensome. In addition, prior studies have shown that increasing access to tenant-rights information and mental health resources alleviates stress among financially constrained households [43]. Our findings thus suggest that policies aiming to reduce both the objective cost burden and the subjective sense of housing stress could be particularly relevant for lower-income renters, although future policy evaluations are required to directly assess their effectiveness.

Our findings also have important implications for evaluating housing policies, including housing voucher programs. As highlighted in a number of diverse prior studies [24, 25, 29, 30], housing affordability is not solely an objective measure of rent-to-income ratios, as subjective experiences of burden and neighborhood conditions play a central role in shaping mental health. If the ultimate goal of housing policy is to enhance the overall quality of life and health, policy evaluations should not be limited to changes in objective metrics (e.g., reducing the RIR from 30 to 25%). Rather, they should incorporate improvements in housing and neighborhood quality to alleviate perceived housing burdens and contribute to better mental health outcomes.

This study has some limitations. First, we used household and housing characteristics measured in 2022 as independent variables, and mental health outcomes measured in 2023 as dependent variables. While this time lag reduced the risk of reverse causality, it also limited our ability to capture changes in independent variables, such as improvements in housing conditions (e.g., bathroom renovations), which may have occurred between the two time points.

Second, our main analyses excluded individuals who relocated between 2022 and 2023, as the housing unit on which affordability was measured would not correspond to the dwelling environment experienced at the time the mental health outcomes were assessed. This restriction helped reduce exposure misclassification but might have introduced selection bias, as cost-burdened households are more likely to experience involuntary moves that may themselves exacerbate mental health concerns. Supplemental analyses including movers (Tables S14-S16 of the Supplementary materials) revealed attenuated associations, likely due to measurement error arising when housing affordability in 2022 does not reflect the housing circumstances experienced in 2023. Thus, our estimates likely represent the conservative effects among households with stable residential contexts.

Third, unobserved individual characteristics, such as personality traits, and prior diagnoses of anxiety or depression may have influenced both perceptions of housing burden and susceptibility to mental distress; however, we were unable to account for these factors. It is also possible that subjective perceptions of housing burden partially reflect the broader financial strain among low-income households, a distinction that future research should examine more directly. Moreover, despite adjusting for a broad set of covariates, residual confounding is unavoidable in observational survey data. As such, the associations reported herein should not be interpreted as causal effects. Future studies using panel data and fixed-effects models may help mitigate these concerns and provide a more rigorous assessment of the relationship between objective and subjective affordability and mental health. Finally, the measurement tools used in this study had limitations. First, the perceived housing cost burden was assessed using a single binary question. While this approach is partly driven by data constraints and is consistent with prior studies [21, 30] that employ binary or simple categorical measures, it nevertheless cannot fully capture the complexity of subjective perceptions. Second, mental health outcomes were not measured using validated instruments such as the DASS-21 or the Kessler Psychological Distress Scale. Therefore, more comprehensive mental health assessments and refined measures of subjective burden will enhance future research.

## Conclusion

This study explored the multidimensional nature of housing affordability and shed light on the complex interplay between housing costs and mental health. Consistent with prior research, the results indicated that policy interventions addressing the mental health consequences of housing cost burdens may be most effective when focused on low-income populations.

Building on the present study, future research should further investigate the underlying motivations and adaptation processes behind high-cost housing choices among higher-income groups as well as the structural vulnerabilities that exacerbate the housing and mental health challenges faced by lower-income households.

## Supplementary Information

Below is the link to the electronic supplementary material.

Supplementary file1 (DOCX 63 KB)

## Data Availability

The data used in this study are publicly accessible through Korea Labor Institute.

## Footnotes

1 Although “housing affordability” is commonly used as a broad conceptual term, we adopted the term “housing unaffordability” in the present study as our measure reflects the degree to which housing costs impose a financial burden.

2 Detailed rationales for the inclusion of all covariates are provided in Table S1 of the Supplementary materials.

3 Weighted descriptive statistics are further presented in Tables S2 and S3 of the Supplementary materials.

4 We further provide unadjusted associations for each affordability metric. Unadjusted results clarify the crude associations between each affordability measure and mental health, thereby reducing the risk of obscuring potential mediating pathways. Please refer to Table S4 in the Supplementary materials for detailed results.
