# Supplementary material for: The Dynamic Relationship Between Objective and Subjective Housing Affordability and Mental Health
Source: J Urban Health. 2026 Mar 26;103(2):309–22. doi: 10.1007/s11524-026-01068-0 (PMC13235675; doi:10.1007/s11524-026-01068-0)
Supplement: Supplementary file 1 — Supplementary file1 (DOCX 63 KB) [file 11524_2026_1068_MOESM1_ESM.docx]

**Supplementary Materials**

**Table S1. Conceptual links between covariates, housing affordability, and mental health**

| **Covariate** | **Relationship to Exposure (Housing Affordability)** | **Relationship to Outcome (Mental Health)** |
| --- | --- | --- |
| Seoul Metropolitan Area | The Seoul metropolitan area has the highest housing costs in Korea; residents face greater affordability challenges and rent burden. | Urban stressors such as crowding and noise are associated with higher stress, but better access to social services |
| Education | Higher education → higher income potential → better affordability and higher housing expectations | Education is associated with better mental health literacy coping resources, and health-promoting behaviors |
| Age | Young renters with less wealth accumulation and career instability → worse affordability  Old renters with fixed income and longer tenure → varying affordability | Younger adults face life transition stress while older adults show resilience, but vulnerability to chronic conditions |
| Marital Status | Married: Dual income and shared expenses may improve affordability, but also increase housing space requirements | Marriage can offer support, but also create role and financial stress |
| Sex | Gender wage gap → Women face greater affordability challenges; single mothers particularly vulnerable | Women report higher depression and stress, partly due to structural constraints, such as care-work inequalities and labor market discrimination |
| Number of Children | More children → greater housing space needs and expenses → worse affordability | Children can be both a stressor and source of meaning |
| Number of Household Members | More people may pool income (positive) but also increase space needs and expenses (negative) | Larger households introduce more social support but also greater risk of conflict and crowding-related stress |
| Housing Type | Apartments in Korea are considered a premium housing, resulting in higher rents, but better amenities and locations | Apartments offer better living conditions → better mental health |

**Note:** All covariates were measured at baseline (2022) to establish temporal precedence. While we controlled for key sociodemographic and housing characteristics, some potential confounders (e.g., personality traits, social networks, neighborhood quality) were not available in our dataset. The temporal ordering of variables (covariates measured at t=2022, outcomes at t=2023) helps to establish temporal precedence and reduce concerns about reverse causation.

**Table S2.** **Weighted summary statistics by objective affordability**

| **Variable** | **Objectively**  **Affordable (RIR ≥30)** | **Objectively unaffordable (RIR <30)** | **P-value** |
| --- | --- | --- | --- |
|  | (N = 2,414) | (N = 541) |  |
| Subjective unaffordability | 0.37 (0.48) | 0.48 (0.5) | 0.040 |
| Stress | 4.42 (1.92) | 4.35 (2.12) | 0.802 |
| Depression | 3.34 (2.07) | 3.41 (1.85) | 0.685 |
| Seoul metropolitan area | 0.54 (0.5) | 0.74 (0.44) | 0.000 |
| Education | 0.27 (0.44) | 0.39 (0.49) | 0.029 |
| Age | 55.02 (15.31) | 59 (16.49) | 0.008 |
| Marriage | 1.8 (0.4) | 1.82 (0.38) | 0.64 |
| Sex | 1.29 (0.45) | 1.36 (0.48) | 0.132 |
| Number of kids | 0.38 (0.77) | 0.31 (0.67) | 0.151 |
| Number of household members | 2.36 (1.28) | 2.12 (1.15) | 0.011 |
| Housing type | 0.45 (0.5) | 0.55 (0.5) | 0.064 |

Note: Values present weighted means and standard deviations using survey weights. P-values were obtained from survey-weighted tests comparing group means or proportions. RIR, rent-to-income ratio

**Table S3.** **Weighted summary statistics by subjective affordability**

| **Variable** | **Subjectively affordable** | **Subjectively unaffordable** | **P-value** |
| --- | --- | --- | --- |
|  | (N = 1,754) | (N = 1,201) |  |
| Objective unaffordability  (RIR ≥30) | 0.17 (0.37) | 0.24 (0.43) | 0.043 |
| Stress | 4.32 (1.94) | 4.54 (1.98) | 0.243 |
| Depression | 3.22 (2.06) | 3.56 (1.97) | 0.043 |
| Seoul metropolitan area | 0.59 (0.49) | 0.57 (0.49) | 0.631 |
| Education | 0.33 (0.47) | 0.24 (0.43) | 0.018 |
| Age | 54.19 (15.48) | 58.28 (15.54) | 0.000 |
| Marriage | 1.81 (0.39) | 1.81 (0.39) | 0.97 |
| Sex | 1.25 (0.44) | 1.38 (0.49) | 0.000 |
| Number of kids | 0.44 (0.8) | 0.24 (0.64) | 0.000 |
| Number of household members | 2.42 (1.28) | 2.16 (1.21) | 0.000 |
| Housing type | 0.47 (0.5) | 0.47 (0.5) | 0.931 |

Note: Values present weighted means and standard deviations using survey weights. P-values were obtained from survey-weighted tests comparing group means or proportions. RIR, rent-to-income ratio

**Table S4. Unadjusted associations between housing affordability and mental health**

|  | **Entire Observations** | | | | **Bottom 40%** | | | | **Top 60%** | | | |
| --- | --- | --- | --- | --- | --- | --- | --- | --- | --- | --- | --- | --- |
| **Variable** | **Stress** | | **Depressive Symptoms** | | **Stress** | | **Depressive Symptoms** | | **Stress** | | **Depressive Symptoms** | |
|  | Model 1 | Model 2 | Model 3 | Model 4 | Model 5 | Model 6 | Model 7 | Model 8 | Model 9 | Model 10 | Model 11 | Model 12 |
| Objective unaffordability | -0.066 (0.091) |  | 0.071 (0.094) |  | 0.102 (0.125) |  | -0.141  (0.131) |  | -0.293*  (0.167) |  | -0.309* (0.166) |  |
| Subjective unaffordability |  | 0.224*** (0.074) |  | 0.342*** (0.076) |  | 0.198* (0.115) |  | 0.060  (0.120) |  | 0.218**  (0.101) |  | 0.245** (0.101) |
| Intercept | 4.420*** (0.040) | 4.319*** (0.046) | 3.342*** (0.042) | 3.222*** (0.048) | 4.339*** (0.069) | 4.268*** (0.082) | 3.876***  (0.072) | 3.803***  (0.086) | 4.458  (0.050) | 4.361  (0.058) | 3.086*** (0.049) | 2.979*** (0.057) |
| Observations | 2955 | 2955 | 2955 | 2955 | 1150 | 1150 | 1150 | 1150 | 1724 | 1724 | 1724 | 1724 |
| Adjusted R² | 0.000 | 0.003 | 0.000 | 0.007 | 0.001 | 0.003 | 0.001 | 0.002 | 0.002 | 0.002 | 0.002 | 0.003 |

Note: *p<0.1; **p<0.05; ***p<0.01. The robust standard error is shown within parentheses.

**Table S5. Association between affordability and mental health, using a 25% rent-to-income threshold**

| **Variable** | **Stress** | | **Depressive symptom** | |
| --- | --- | --- | --- | --- |
|  | **Model 1** | **Model 2** | **Model 3** | **Model 4** |
| Objective unaffordability | -0.089  (0.086) | -0.254**  (0.114) | -0.071  (0.086) | -0.217*  (0.115) |
| Subjective unaffordability | 0.261***  (0.075) | 0.164*  (0.087) | 0.183**  (0.076) | 0.097  (0.088) |
| Seoul metropolitan area | -0.098  (0.075) | -0.084  (0.075) | -0.081  (0.075) | -0.069  (0.075) |
| Education | -0.019  (0.086) | -0.036  (0.086) | -0.006  (0.086) | -0.022  (0.087) |
| Age | -0.006**  (0.003) | -0.006**  (0.003) | 0.027***  (0.003) | 0.027***  (0.003) |
| Marriage | 0.177  (0.130) | 0.175  (0.130) | -0.068  (0.131) | -0.070  (0.131) |
| Sex | -0.105  (0.084) | -0.115  (0.084) | 0.383***  (0.085) | 0.374***  (0.085) |
| Number of Kid | -0.143**  (0.067) | -0.135**  (0.067) | 0.009  (0.067) | 0.016  (0.067) |
| Number of household members | 0.091**  (0.045) | 0.090**  (0.045) | -0.026  (0.045) | -0.028  (0.045) |
| Housing type | -0.294***  (0.076) | -0.284***  (0.076) | -0.379***  (0.076) | -0.370***  (0.076) |
| Objective × Subjective unaffordability |  | 0.370**  (0.168) |  | 0.326*  (0.169) |
| Constant | 4.713***  (0.218) | 4.761***  (0.219) | 1.658***  (0.219) | 1.700***  (0.220) |
| Observations | 2,955 | 2,955 | 2,955 | 2,955 |
| Adjusted R² | 0.011 | 0.013 | 0.068 | 0.069 |

Note: *p<0.1; **p<0.05; ***p<0.01. The robust standard error is shown within parentheses.

**Table S6. Association between affordability and mental health using a 35% rent-to-income threshold**

| **Variable** | **Stress** | | **Depressive symptom** | |
| --- | --- | --- | --- | --- |
|  | **Model 1** | **Model 2** | **Model 3** | **Model 4** |
| Objective unaffordability | 0.078  (0.102) | -0.300**  (0.136) | 0.063  (0.102) | -0.230*  (0.137) |
| Subjective unaffordability | 0.251***  (0.075) | 0.115  (0.082) | 0.175**  (0.075) | 0.069  (0.082) |
| Seoul metro area | -0.122  (0.074) | -0.087  (0.075) | -0.100  (0.075) | -0.074  (0.075) |
| Education | -0.038  (0.086) | -0.075  (0.087) | -0.022  (0.087) | -0.050  (0.087) |
| Age | -0.007**  (0.003) | -0.007**  (0.003) | 0.026***  (0.003) | 0.026***  (0.003) |
| Marriage | 0.174  (0.130) | 0.160  (0.130) | -0.071  (0.131) | -0.082  (0.130) |
| Sex | -0.108  (0.084) | -0.133  (0.084) | 0.381***  (0.085) | 0.361***  (0.085) |
| Number of Kid | -0.148**  (0.067) | -0.129*  (0.067) | 0.004  (0.067) | 0.019  (0.067) |
| Number of household members | 0.098**  (0.045) | 0.089**  (0.045) | -0.021  (0.045) | -0.028  (0.045) |
| Housing type | -0.305***  (0.076) | -0.291***  (0.075) | -0.387***  (0.076) | -0.377***  (0.076) |
| Objective × Subjective unaffordability |  | 0.834**  (0.200) |  | 0.646***  (0.202) |
| Constant | 4.726***  (0.218) | 4.814***  (0.219) | 1.669***  (0.219) | 1.737***  (0.220) |
| Observations | 2,955 | 2,955 | 2,955 | 2,955 |
| Adjusted R² | 0.011 | 0.017 | 0.068 | 0.071 |

Note: *p<0.1; **p<0.05; ***p<0.01. The robust standard error is shown within parentheses.

# **Table S7. Associations between housing affordability and mental health, using a 50% rent-to-income threshold**

| **Variable** | **Stress** | | **Depressive symptoms** | |
| --- | --- | --- | --- | --- |
|  | **Model 1** | **Model 2** | **Model 3** | **Model 4** |
| Objective unaffordability | 0.344*** (0.129) | -0.308* (0.165) | 0.105 (0.129) | -0.379** (0.166) |
| Subjective unaffordability | 0.254*** (0.075) | 0.106 (0.078) | 0.177** (0.075) | 0.067 (0.079) |
| Seoul metro area | -0.150** (0.075) | -0.130* (0.074) | -0.104 (0.075) | -0.090 (0.075) |
| Education | -0.042 (0.086) | -0.094 (0.085) | -0.018 (0.086) | -0.057 (0.086) |
| Age | -0.007** (0.003) | -0.008** (0.003) | 0.026*** (0.003) | 0.026*** (0.003) |
| Marriage | 0.177 (0.130) | 0.174 (0.129) | -0.070 (0.131) | -0.072 (0.130) |
| Sex | -0.097 (0.084) | -0.097 (0.084) | 0.385*** (0.085) | 0.384*** (0.084) |
| Number of Kid | -0.153** (0.067) | -0.144** (0.066) | 0.004 (0.067) | 0.010 (0.067) |
| Number of household members | 0.106** (0.045) | 0.098** (0.045) | -0.019 (0.045) | -0.026 (0.045) |
| Housing type | -0.305*** (0.075) | -0.283*** (0.075) | -0.385*** (0.076) | -0.369*** (0.076) |
| Objective × Subjective unaffordability |  | 1.589*** (0.254) |  | 1.179*** (0.256) |
| Constant | 4.548*** (0.219) | 4.643*** (0.218) | 1.734*** (0.221) | 1.805*** (0.220) |
| Observations | 2,955 | 2,955 | 2,955 | 2,955 |
| Adjusted R² | 0.014 | 0.026 | 0.068 | 0.075 |

Note: *p<0.1; **p<0.05; ***p<0.01. Robust standard errors are shown within parentheses.

# **Table S8. Associations between housing affordability and mental health (bottom 40% income group, 50% threshold)**

| **Variable** | **Stress** | | **Depressive symptoms** | |
| --- | --- | --- | --- | --- |
|  | **Model 1** | **Model 2** | **Model 3** | **Model 4** |
| Objective unaffordability | 0.600*** (0.178) | -0.380 (0.244) | 0.070 (0.189) | -0.622** (0.260) |
| Subjective unaffordability | 0.162 (0.114) | -0.081 (0.120) | 0.007 (0.121) | -0.165 (0.128) |
| Seoul metro area | -0.434*** (0.118) | -0.423*** (0.117) | -0.284** (0.125) | -0.275** (0.124) |
| Education | 0.235 (0.159) | 0.115 (0.158) | -0.168 (0.168) | -0.253 (0.169) |
| Age | -0.014*** (0.005) | -0.014*** (0.005) | 0.017*** (0.005) | 0.017*** (0.005) |
| Marriage | 0.580*** (0.188) | 0.554*** (0.185) | 0.140 (0.199) | 0.122 (0.197) |
| Sex | -0.281** (0.119) | -0.281** (0.117) | 0.148 (0.126) | 0.148 (0.125) |
| Number of Kid | -0.200 (0.200) | -0.128 (0.198) | -0.071 (0.212) | -0.020 (0.211) |
| Number of household members | 0.162* (0.092) | 0.155* (0.091) | 0.123 (0.097) | 0.119 (0.097) |
| Housing type | -0.310*** (0.116) | -0.286** (0.114) | -0.101 (0.123) | -0.084 (0.122) |
| Objective × Subjective unaffordability |  | 1.940*** (0.335) |  | 1.371*** (0.357) |
| Constant | 4.590*** (0.331) | 4.730*** (0.328) | 2.310*** (0.350) | 2.408*** (0.349) |
| Observations | 1,150 | 1,150 | 1,150 | 1,150 |
| Adjusted R² | 0.049 | 0.076 | 0.030 | 0.041 |

Note: *p<0.1; **p<0.05; ***p<0.01. Robust standard errors are shown within parentheses.

# **Table S9. Associations between housing affordability and mental health (top 60% income group, 50% threshold)**

| **Variable** | **Stress** | | **Depressive symptoms** | |
| --- | --- | --- | --- | --- |
|  | **Model 1** | **Model 2** | **Model 3** | **Model 4** |
| Objective unaffordability | -0.274 (0.354) | -0.286 (0.416) | -0.044 (0.344) | -0.303 (0.404) |
| Subjective unaffordability | 0.180* (0.103) | 0.179* (0.104) | 0.136 (0.121) | 0.120 (0.128) |
| Seoul metro area | 0.024 (0.099) | 0.024 (0.099) | 0.052 (0.100) | 0.050 (0.101) |
| Education | -0.228** (0.106) | -0.228** (0.106) | -0.018 (0.103) | -0.015 (0.103) |
| Age | -0.001 (0.005) | -0.001 (0.005) | 0.027*** (0.005) | 0.026*** (0.005) |
| Marriage | -0.222 (0.187) | -0.221 (0.187) | -0.116 (0.116) | -0.103 (0.116) |
| Sex | -0.023 (0.125) | -0.022 (0.125) | 0.414*** (0.121) | 0.418*** (0.121) |
| Number of Kid | -0.047 (0.081) | -0.047 (0.081) | 0.021 (0.079) | 0.018 (0.079) |
| Number of household members | 0.080 (0.061) | 0.080 (0.061) | 0.005 (0.059) | 0.005 (0.059) |
| Housing type | -0.254** (0.101) | -0.253** (0.101) | -0.513*** (0.059) | -0.511*** (0.059) |
| Objective × Subjective unaffordability |  | 0.041 (0.781) |  | 0.928 (0.099) |
| Constant | 4.829*** (0.328) | 4.829*** (0.329) | 1.596*** (0.319) | 1.581*** (0.320) |
| Observations | 1,724 | 1,724 | 1,724 | 1,724 |
| Adjusted R² | 0.008 | 0.007 | 0.046 | 0.046 |

Note: *p<0.1; **p<0.05; ***p<0.01. Robust standard errors are shown within parentheses.

**Table S10. Post-hoc power analysis for the interaction effects**

| **Model** | Observations | **Outcome** | **βeta** | **Standard Errors** | **t-statistics** | **f²** | **Achieved Power (%)** |
| --- | --- | --- | --- | --- | --- | --- | --- |
| **Full Sample** | 2,955 | Stress | 0.597*** | 0.184 | 3.25 | 0.0036 | 90.2 |
|  |  | Depression | 0.411** | 0.185 | 2.22 | 0.0017 | 60.5 |
| **Bottom 40% Income Group** | 1,150 | Stress | 0.451** | 0.206 | 2.19 | 0.0042 | 59.4 |
|  |  | Depression | 0.449* | 0.269 | 1.67 | 0.0024 | 38.9 |
| **Top 60% Income Group** | 1,724 | Stress | 0.457 | 0.363 | 1.26 | 0.0009 | 24.3 |
|  |  | Depression | 0.034 | 0.353 | 0.10 | <0.0001 | 5.1 |

*Note: Statistical power was calculated using observed effect sizes, sample sizes, and model parameters* *(α=0.05, two-tailed) . Cohen's f² effect sizes were computed as f² = t²/(N - k - 1), where k = 11 predictors. All groups had minimum detectable effects < 0.02, indicating adequate sensitivity. *p<0.10; **p<0.05; ***p<0.01.*

Post-hoc power analyses indicated an adequate statistical power to detect interaction terms in the full sample (Stress: 90.2%; Depression: 60.5%). In the bottom 40% income group, interaction patterns were observed, despite only achieving a relatively modest power (38.9–59.4%), suggesting that the estimated associations may be conservative with respect to their underlying magnitude. Across all income groups, the minimum detectable effect sizes were well below the conventional thresholds for small effects (Cohen’s f² = 0.02), indicating that stratified analyses were conducted with samples that were generally sufficient to detect modest associations. In contrast, for the top 60% income group, the interaction term for depressive symptoms was very small (β = 0.034), with the point estimate substantially smaller than its standard error, a pattern consistent with a negligible association rather than limited statistical power.

The interaction term for stress warrants a more cautious interpretation. In the top 60% income group, the point estimate for the interaction (β = 0.457) was similar in magnitude to that observed in the bottom 40% group (β = 0.451), but was accompanied by a substantially larger standard error (SE = 0.363 vs. 0.206), thus resulting in a statistically non-significant estimate. Given the low power achieved in this subgroup (24.3%), we cannot fully exclude the possibility of a modest interaction. Nevertheless, several considerations indicate that any such association is likely weaker or less salient among higher-income households. First, the corresponding interaction for depressive symptoms was close to zero. Second, theoretical perspectives emphasize the lower vulnerability to housing-related stressors among higher-income groups. Third, the larger sample size in the top 60% income group (N = 1,724 vs. 1,150) would be expected to provide adequate precision, if the interaction were comparable in magnitude. Taken together, these patterns are more consistent with income-related heterogeneity in the salience of housing affordability for mental health, rather than differences driven solely by statistical power.

**Table S11. Associations between housing affordability and mental health (bottom 20% income group)**

| **Variable** | **Stress** | | **Depressive symptoms** | |
| --- | --- | --- | --- | --- |
|  | **Model 1** | **Model 2** | **Model 3** | **Model 4** |
| Objective unaffordability | -0.375** (0.175) | -0.750*** (0.245) | -0.391** (0.191) | -0.484* (0.267) |
| Subjective unaffordability | 0.101 (0.156) | -0.156 (0.195) | 0.395** (0.169) | 0.332 (0.213) |
| Seoul metro area | -0.552*** (0.172) | -0.527*** (0.172) | -0.304 (0.187) | -0.298 (0.187) |
| Education | 0.776*** (0.285) | 0.755*** (0.285) | 0.628** (0.310) | 0.622** (0.311) |
| Age | -0.007 (0.007) | -0.006 (0.007) | 0.021*** (0.008) | 0.021*** (0.008) |
| Marriage | 0.606** (0.253) | 0.602** (0.252) | 0.097 (0.275) | 0.096 (0.275) |
| Sex | -0.001 (0.175) | -0.035 (0.175) | 0.036 (0.190) | 0.028 (0.191) |
| Number of Kid | 0.369 (0.485) | 0.357 (0.483) | -0.121 (0.527) | -0.124 (0.528) |
| Number of household members | 0.304* (0.156) | 0.333** (0.156) | 0.199 (0.169) | 0.206 (0.170) |
| Housing type | 0.084 (0.156) | 0.084 (0.156) | 0.166 (0.170) | 0.166 (0.170) |
| Objective × Subjective unaffordability |  | 0.699** (0.321) |  | 0.174 (0.350) |
| Constant | 4.235*** (0.547) | 4.253*** (0.545) | 2.237*** (0.594) | 2.241*** (0.595) |
| Observations | 575 | 575 | 575 | 575 |
| Adjusted R² | 0.053 | 0.060 | 0.042 | 0.041 |

Note: *p<0.1; **p<0.05; ***p<0.01. The robust standard error is shown within parentheses.

**Table S12.** **Associations between housing affordability and mental health (middle 60% income group)**

| **Variable** | **Stress** | | **Depressive symptoms** | |
| --- | --- | --- | --- | --- |
|  | **Model 1** | **Model 2** | **Model 3** | **Model 4** |
| Objective unaffordability | 0.280* (0.148) | -0.015 (0.212) | 0.008 (0.147) | -0.202 (0.211) |
| Subjective unaffordability | 0.130 (0.100) | 0.051 (0.108) | -0.048 (0.100) | -0.104 (0.107) |
| Seoul metro area | 0.062 (0.099) | 0.072 (0.099) | -0.022 (0.098) | -0.015 (0.098) |
| Education | -0.178 (0.116) | -0.201* (0.116) | -0.196* (0.115) | -0.213* (0.116) |
| Age | -0.010** (0.005) | -0.010** (0.005) | 0.021*** (0.005) | 0.021*** (0.005) |
| Marriage | -0.037 (0.173) | -0.044 (0.173) | -0.083 (0.173) | -0.088 (0.173) |
| Sex | -0.174 (0.109) | -0.186* (0.109) | 0.423*** (0.108) | 0.415*** (0.109) |
| Number of Kid | -0.304*** (0.097) | -0.282*** (0.097) | -0.221** (0.096) | -0.206** (0.097) |
| Number of household members | 0.246*** (0.063) | 0.239*** (0.063) | 0.209*** (0.063) | 0.204*** (0.063) |
| Housing type | -0.477*** (0.104) | -0.458*** (0.105) | -0.418*** (0.104) | -0.404*** (0.104) |
| Objective × Subjective unaffordability |  | 0.559* (0.286) |  | 0.397 (0.285) |
| Constant | 4.930*** (0.288) | 4.983*** (0.289) | 1.582*** (0.287) | 1.619*** (0.288) |
| Observations | 1,726 | 1,726 | 1,726 | 1,726 |
| Adjusted R² | 0.027 | 0.028 | 0.048 | 0.049 |

Note: *p<0.1; **p<0.05; ***p<0.01. The robust standard error is shown within parentheses.

**Table S13. Associations between housing affordability and mental health (top 20% income group)**

| **Variable** | **Stress** | | **Depressive symptoms** | |
| --- | --- | --- | --- | --- |
|  | **Model 1** | **Model 2** | **Model 3** | **Model 4** |
| Objective unaffordability | 0.025 (0.280) | -0.140 (0.307) | -0.140 (0.259) | -0.088 (0.285) |
| Subjective unaffordability | 0.666*** (0.185) | 0.605*** (0.191) | 0.395** (0.171) | 0.414** (0.177) |
| Seoul metro area | -0.189 (0.166) | -0.183 (0.166) | 0.163 (0.154) | 0.161 (0.154) |
| Education | -0.012 (0.162) | -0.023 (0.162) | 0.226 (0.149) | 0.230 (0.150) |
| Age | -0.009 (0.010) | -0.010 (0.010) | 0.011 (0.009) | 0.011 (0.009) |
| Marriage | 0.158 (0.554) | 0.152 (0.554) | -0.100 (0.512) | -0.098 (0.513) |
| Sex | -0.247 (0.255) | -0.245 (0.255) | 0.357 (0.236) | 0.357 (0.236) |
| Number of Kid | 0.051 (0.119) | 0.050 (0.119) | 0.131 (0.110) | 0.131 (0.110) |
| Number of household members | -0.154 (0.100) | -0.147 (0.100) | -0.318*** (0.093) | -0.320*** (0.093) |
| Housing type | -0.001 (0.174) | -0.002 (0.174) | -0.362** (0.161) | -0.362** (0.161) |
| Objective × Subjective unaffordability |  | 0.883 (0.687) |  | -0.276 (0.636) |
| Constant | 5.341*** (0.725) | 5.389*** (0.726) | 2.872*** (0.670) | 2.857*** (0.672) |
| Observations | 573 | 573 | 573 | 573 |
| Adjusted R² | 0.011 | 0.012 | 0.048 | 0.047 |

Note: *p<0.1; **p<0.05; ***p<0.01. The robust standard error is shown within parentheses.

**Table S14. Associations between housing affordability and mental health in the full sample, including movers**

| **Variable** | **Stress** | | **Depressive symptoms** | |
| --- | --- | --- | --- | --- |
|  | **Model 1** | **Model 2** | **Model 3** | **Model 4** |
| Objective unaffordability | 0.027 (0.069) | -0.029 (0.092) | -0.037 (0.070) | -0.029 (0.094) |
| Subjective unaffordability | 0.089* (0.054) | 0.065 (0.060) | -0.007 (0.055) | -0.003 (0.061) |
| Seoul metropolitan area | -0.063 (0.053) | -0.058 (0.053) | 0.044 (0.054) | 0.044 (0.054) |
| Education | -0.296*** (0.061) | -0.302*** (0.061) | -0.146** (0.062) | -0.145** (0.062) |
| Age | -0.004 (0.002) | -0.004 (0.002) | 0.026*** (0.003) | 0.026*** (0.003) |
| Marriage | 0.185* (0.109) | 0.181* (0.109) | 0.005 (0.112) | 0.006 (0.112) |
| Sex | -0.071 (0.064) | -0.076 (0.065) | 0.285*** (0.066) | 0.286*** (0.066) |
| Number of kids | -0.073*  (0.042) | -0.070*  (0.042) | 0.043 (0.042) | 0.043 (0.043) |
| Number of household members | -0.01 (0.03) | -0.011 (0.03) | -0.108*** (0.03) | -0.108*** (0.03) |
| Housing type | -0.201*** (0.054) | -0.198*** (0.054) | -0.395*** (0.055) | -0.396*** (0.055) |
| Objective × subjective unaffordability |  | 0.126 (0.136) |  | -0.018 (0.139) |
| Constant | 4.730*** (0.174) | 4.741*** (0.174) | 1.922*** (0.178) | 1.920*** (0.178) |
| Observations | 5313 | 5313 | 5313 | 5313 |
| Adjusted R² | 0.010 | 0.010 | 0.068 | 0.067 |

Note: *p<0.1; **p<0.05; ***p<0.01. The robust standard error is shown within parentheses

**Table S15. Associations between housing affordability and mental health among low-income renters (bottom 40%), including movers**

| **Variable** | **Stress** | | **Depressive symptoms** | |
| --- | --- | --- | --- | --- |
|  | **Model 1** | **Model 2** | **Model 3** | **Model 4** |
| Objective unaffordability | -0.057  (0.099) | 0.102 (0.148) | -0.148 (0.101) | -0.052 (0.152) |
| Subjective unaffordability | 0.041  (0.084) | 0.111 (0.097) | -0.162* (0.086) | -0.119 (0.099) |
| Seoul metropolitan area | -0.180** (0.084) | -0.192** (0.085) | -0.155* (0.086) | -0.162* (0.087) |
| Education | 0.078  (0.109) | 0.098 (0.110) | -0.155 (0.112) | -0.143 (0.113) |
| Age | -0.009** (0.004) | -0.009*** (0.004) | 0.020*** (0.004) | 0.020*** (0.004) |
| Marriage | 0.350** (0.151) | 0.357** (0.152) | 0.155 (0.155) | 0.159 (0.155) |
| Sex | -0.068  (0.088) | -0.053 (0.088) | 0.290*** (0.090) | 0.300*** (0.090) |
| Number of kids | -0.058  (0.098) | -0.077 (0.099) | 0.147 (0.101) | 0.136 (0.102) |
| Number of household members | 0.093*  (0.055) | 0.098* (0.055) | -0.060 (0.056) | -0.057 (0.056) |
| Housing type | -0.229*** (0.087) | -0.229*** (0.087) | -0.104 (0.089) | -0.103 (0.089) |
| Objective × subjective unaffordability |  | -0.281 (0.195) |  | -0.169 (0.200) |
| Constant | 4.724*** (0.261) | 4.684*** (0.262) | 2.260*** (0.267) | 2.236*** (0.268) |
| Observations | 2108 | 2108 | 2108 | 2108 |
| Adjusted R² | 0.011 | 0.011 | 0.042 | 0.042 |

Note: *p<0.1; **p<0.05; ***p<0.01. The robust standard error is shown within parentheses

**Table S16. Associations between housing affordability and mental health among higher-income renters (top 60%), including movers**

| **Variable** | **Stress** | | **Depressive symptoms** | |
| --- | --- | --- | --- | --- |
|  | **Model 1** | **Model 2** | **Model 3** | **Model 4** |
| Objective unaffordability | -0.039 (0.123) | -0.105 (0.140) | 0.022 (0.125) | 0.047 (0.142) |
| Subjective unaffordability | 0.020 (0.074) | -0.001 (0.077) | -0.016 (0.075) | -0.008 (0.078) |
| Seoul metropolitan area | 0.036 (0.069) | 0.038 (0.069) | 0.260*** (0.070) | 0.260*** (0.070) |
| Education | -0.504*** (0.074) | -0.507*** (0.075) | -0.159** (0.076) | -0.158** (0.076) |
| Age | 0.008** (0.004) | 0.008** (0.004) | 0.030*** (0.004) | 0.030*** (0.004) |
| Marriage | -0.185 (0.183) | -0.181 (0.183) | -0.061 (0.186) | -0.062 (0.186) |
| Sex | -0.125 (0.105) | -0.122 (0.105) | 0.003 (0.106) | 0.002 (0.106) |
| Number of kids | 0.052 (0.051) | 0.052 (0.051) | 0.038 (0.052) | 0.037 (0.052) |
| Number of household members | -0.110*** (0.041) | -0.108*** (0.042) | -0.100** (0.042) | -0.101** (0.042) |
| Housing type | -0.141** (0.071) | -0.140** (0.071) | -0.542*** (0.072) | -0.542*** (0.072) |
| Objective × subjective unaffordability |  | 0.269 (0.276) |  | -0.101 (0.280) |
| Constant | 4.755*** (0.270) | 4.752*** (0.270) | 1.963*** (0.274) | 1.964*** (0.274) |
| Observations | 3081 | 3081 | 3081 | 3081 |
| Adjusted R² | 0.027 | 0.027 | 0.064 | 0.064 |

Note: *p<0.1; **p<0.05; ***p<0.01. The robust standard error is shown within parentheses
